# Modulation of the endocannabinoid system reduces inflammatory signalling in canine mammary carcinoma cells

**DOI:** 10.1002/vro2.70034

**Published:** 2026-04-29

**Authors:** Gianluca Antonio Franco, Ylenia Marino, Claudia Rifici, Roberta Fusco, Davide di Paola, Salvatore Cuzzocrea, Giuseppe Catone, Cecilia Vullo, Enrico Gugliandolo

**Affiliations:** ^1^ Department of Veterinary Science University of Messina Messina Italy; ^2^ Department of Chemical Biological Pharmaceutical and Environmental Science University of Messina Messina Italy; ^3^ Link University Rome Campus Italy Rome Italy

**Keywords:** cannabidiol, dogs, endocannabinoid receptors, inflammation, mammary tumour, PPAR‐α

## Abstract

**Background:**

Canine mammary carcinoma (CMC) is characterised by a chronic inflammatory microenvironment resembling human breast cancer; however, the upstream regulatory mechanisms driving this phenotype remain unclear. The endocannabinoid system (ECS) has emerged as a potential modulator of inflammation and tumour biology. This study investigated the role of the ECS in CMC and evaluated the anti‐inflammatory effects of cannabidiol (CBD).

**Methods:**

Primary cell cultures were established from surgically excised CMC tissues, with matched normal mammary epithelium used as controls. Basal mRNA expression of ECS‐related receptors (CB1, CB2, transient receptor potential vanilloid 1 [TRPV1], G‐protein‐coupled receptor 55 [GPR55] and peroxisome proliferator‐activated receptor alpha [PPAR‐α]) and inflammatory mediators (COX‐1, COX‐2, interleukin [IL]‐4, IL‐6, IL‐33, IL‐17A, tumour necrosis factor‐alpha [TNF‐α] and LCN2) was assessed by reverse transcription quantitative polymerase chain reaction. Cytokine secretion (IL‐6, IL‐8, TNF‐α and IL‐17A) was quantified by enzyme‐linked immunosorbent assay. Cell viability assays were performed to determine the 24‐h IC_50_ of CBD (32 µM), and sub‐cytotoxic concentrations (3, 10 and 20 µM) were subsequently applied for 24 h.

**Results:**

Canine mammary carcinoma‐derived cells exhibited significant overexpression of ECS receptors (CB1, CB2, TRPV1, GPR55 and PPAR‐α) compared to normal controls. These cells also showed increased secretion of pro‐inflammatory cytokines, including IL‐6, IL‐8, TNF‐α and IL‐17A. Treatment with CBD at 10–20 µM significantly downregulated key inflammatory genes, particularly COX‐2, IL‐6 and TNF‐α, and reduced corresponding cytokine release without compromising cell viability.

**Conclusion:**

The ECS is upregulated in CMC and appears to contribute to the inflammatory tumour microenvironment. Cannabidiol effectively attenuates this inflammatory phenotype at sub‐cytotoxic concentrations, supporting its potential as a therapeutic agent in CMC.

## INTRODUCTION

The canine mammary gland is a tubuloalveolar structure comprising stroma, parenchyma, vessels, ducts and nerves.^1^ The posterior mammary glands are more frequently affected than the anterior glands and may present with one or more nodules.^2^ Breast tumours are among the most common cancers in dogs and account for approximately 25%‒30% of all the tumours recorded in dogs.^3^ Dogs can develop either benign or malignant tumours. Malignant tumours tend to be locally invasive, often fixed to the overlying skin or underlying tissues, and generally grow rapidly.^4^ In contrast, benign tumours are usually small, well circumscribed and slow growing.^2^, ^5^ Metastases most commonly occur in regional lymph nodes and lungs, although the brain, bones and various abdominal organs can also be involved.^6^ Recent research has highlighted the roles of ovarian hormones and certain growth factors in the aetiology of breast tumours.^6^

Dogs are valuable models for studying malignancies such as lymphoma and mammary tumours because their cancers develop spontaneously and share biological features with human disease.^7^ Inflammation plays a crucial role in this process, and rather than combating tumourigenesis, it often potentiates it.^8^ Inflammation leads to cancer through two pathways: an intrinsic pathway driven by genetic changes (e.g., oncogene activation) and an extrinsic pathway caused by chronic inflammation at specific sites.^9^ Both pathways converge to create an inflammatory tumour microenvironment that promotes malignant transformation.^10^, ^11^ Immune cells are continually recruited and activated, thereby perpetuating this inflammatory milieu and fostering tumour development.^12^ Beyond these well‐characterised mechanisms, recent studies have identified endogenous regulatory systems as key modulators of tumour progression and inflammation.

In particular, the endocannabinoid system (ECS) has emerged as a critical modulator of the chronic inflammatory milieu that drives tumour progression.^13^ Previous studies have indicated that endocannabinoid levels rise in proportion to disease severity in breast cancer, suggesting a direct link to tumour progression.^14^ The ECS comprises natural endocannabinoids and their receptors, CB1 and CB2.^15^ When endocannabinoids bind to these receptors, they activate G‐proteins that regulate several intracellular signalling pathways, including the MAPK/ERK, PI3K/Akt and cAMP‐dependent pathways.^16^ Modulation of these pathways can lead to reduced cell proliferation, induction of apoptosis and autophagy, inhibition of angiogenesis, and decreased cell migration and invasion, ultimately contributing to the suppression of tumour progression.^17^ Among the exogenous cannabinoids, cannabidiol (CBD) has garnered significant attention for its ability to interfere with pro‐tumourigenic inflammatory loops and inhibit cancer cell proliferation through interactions with multiple molecular targets, including CB1 and CB2 receptors, transient receptor potential vanilloid (TRPV) channels, G‐protein‐coupled receptor 55 (GPR55) and peroxisome proliferator‐activated receptors (PPARs).^18^ Cannabinoids can thus affect survival, proliferation, invasion and metastasis in tumour cells, exhibiting anti‐proliferative, pro‐apoptotic and anti‐angiogenic properties.^19^, ^20^ Mechanistically, cannabinoids have been shown to induce autophagy and apoptosis, as well as to inhibit vascular endothelial growth factor. Moreover, the ECS exerts anti‐inflammatory and immunomodulatory effects, contributing to its multi‐level action.

Based on these insights, we aimed to investigate whether targeting the ECS using cannabinoids might induce cancer cell death.^21^ In addition to the classical cannabinoid receptors (CB1 and CB2), this study investigated the role of other ECS's receptor, including the peroxisome proliferator‐activated receptor alpha (PPAR‐α). PPAR‐α is a ligand‐activated transcription factor widely recognised for its potent anti‐inflammatory properties and its crucial involvement in regulating cell cycle progression and apoptosis in various malignancies, including breast cancer.^22^, ^23^ Notably, in oncological contexts, PPAR‐α is often co‐expressed or cross‐modulated with the ECS, suggesting a complex interplay that may influence tumour cell fate and the local inflammatory microenvironment.^21^, ^24^, ^25^, ^26^ Based on these findings, we hypothesised that the ECS plays a key role in sustaining the aggressive inflammatory phenotype of canine mammary carcinoma (CMC), and that targeting this system with CBD may mitigate these pro‐tumourigenic processes. Consequently, this study aimed to characterise the expression of ECS receptors, including the involvement of ECS's receptors such as PPAR‐α, and inflammatory markers in primary CMC cell cultures.

## MATERIALS AND METHODS

### Samples

Mammary gland lesions were surgically excised for therapeutic purposes from privately owned dogs at the Veterinary Teaching Hospital of Messina. Surplus tissue was donated to the study after informed consent was obtained from each owner. Three intact female dogs (Beagle, 6 years; Rottweiler, 10 years; and German Shepherd dog, 13 years) met the inclusion criteria: (i) primary tumour, (ii) no prior chemotherapy or anti‐inflammatory medication, and (iii) histological confirmation of mixed‐type carcinoma with focal comedocarcinoma (WHO classification, grade II), verified independently by two board‐certified pathologists. Immediately after excision, specimens were divided: one fragment was fixed in 10% neutral‐buffered formalin (<30 min post‐surgery) for histopathology, while a second fragment was placed on ice in Dulbecco's phosphate‐buffered saline (PBS) with penicillin (100 IU/mL) and streptomycin (100 µg/mL) and processed for primary cell isolation within 2 h. Control tissue consisted of macroscopically normal mammary epithelium from the adjacent gland of the same dog; when unavailable, histologically normal tissue excised for unrelated reasons in age‐matched bitches was used. All procedures were conducted in accordance with the European Directive 2010/63/EU. Samples were anonymised and coded for downstream analyses.

### Cannabidiol

Cannabidiol (C‐045‐1ML, Cerilliant Sigma‒Aldrich) was dissolved in methanol to prepare the stock solution (1.0 mg/mL). For all experimental applications, final dilutions were adjusted based on the tests performed. The prepared CBD was then stored at 4°C.

### Cell isolation

Surgically excised tumour specimens were immediately placed on ice and trimmed of adipose tissue under aseptic conditions. Tissues were rinsed twice in cold PBS supplemented with antibiotics as previously described, then minced into ∼1 mm^3^ fragments with sterile scalpels. Tissue fragments were mechanically disaggregated by gentle pressing through a 70 µm cell strainer (Becton Dickinson Falcon) into ice‐cold Dulbecco's modified Eagle medium (DMEM, Gibco). The suspension was centrifuged (300 ×*g*, 10 min, 4°C) and the pellet was resuspended in DMEM, supplemented with 10% fetal bovine serum, 2 mM L‐glutamine and antibiotics (100 IU/mL penicillin and 100 µg/mL streptomycin). Cells (1 × 10^5^ cells/cm^2^) were seeded in 60 mm tissue‐culture dishes (Costar, Corning) and incubated at 37°C in a humidified 5% CO_2_ atmosphere. Medium was changed after 24 h to remove non‐adherent debris and thereafter every 48 h. Upon reaching 80%–90% confluence (≈5–7 days), cells were trypsinised (0.05% trypsin‒EDTA, 5 min, 37°C) and sub‐cultured at a 1:2 ratio; passage 5 cells were used for all downstream assays. Cell viability was 95% or more by trypan‐blue exclusion, and cultures were routinely screened for Mycoplasma contamination with a polymerase chain reaction (PCR)‐based kit (Eurofins).

### Histological examination

Tissue fragments were fixed in 10% neutral‐buffered formalin for 24 h at room temperature, routinely processed, and embedded in paraffin. Serial 5 µm sections were cut on a rotary microtome (Leica Microsystems). Paraffin‐embedded tissue sections were first deparaffinised in xylene through three changes of 5 min each. Sections were then rehydrated through a graded ethanol series (100%, 95%, 70% and 50%), with 10 min in 100% and 95% ethanol, followed by 5 min in 70% and 50% ethanol, and finally rinsed in distilled water for 5 min.

Sections were stained with haematoxylin for 5 min and subsequently washed in running tap water until the excess stain was removed. The slides were then counterstained with eosin for 3 min.

After staining, sections were dehydrated through graded ethanol, including rapid washes followed by 95% ethanol for 1 min and two changes of 100% ethanol for 5 min each. Finally, sections were cleared in xylene for 15 min, mounted with a permanent mounting medium, and coverslipped. Histological evaluation was performed independently by two board‐certified veterinary pathologists using a Leica DM2500 microscope (Leica Microsystems).

### Immunofluorescence

Cells (5 × 10^4^ cells/well) were seeded directly onto the optically clear bottoms of standard 12‐well culture plates and grown to 70%–80% confluence. Monolayers were rinsed twice with PBS and fixed in 4% paraformaldehyde/PBS for 15 min at room temperature, permeabilised with 0.1% Triton X‐100 (5 min) and blocked for 1 h in PBS containing 5% bovine serum albumin. Primary antibodies—PPAR‐α (rabbit, ab8934, 1:200) or β‐tubulin‐Alexa Fluor 488 (mouse, MA5‐16308, 1:400) previously validated in dog ^27^—were applied overnight at 4°C. After three PBS washes, wells were incubated for 1 h at room temperature with species‐specific secondary antibodies (goat anti‐rabbit‐Texas red both 1:500 #T‐2767, Thermo Fisher), counterstained with 4′,6‐diamidino‐2‐phenylindole (1 µg/mL, 5 min) and maintained in PBS for imaging. Fluorescence micrographs were captured in‐plate with an EVOS M5000 system (20× and 40× objectives) using identical exposure settings for all conditions.

### Reverse transcription quantitative polymerase chain reaction

Total RNA was extracted from each experimental group with the RNeasy Mini Kit (Qiagen, Cat. #74104) according to the manufacturer's protocols.^28^ After quantifying the RNA by nanodrop, we performed c‐DNA synthesis using the iScript cDNA Synthesis Kit (Bio‐Rad, Cat. #1708891) according to the manufacturer's protocols.^29^ Polymerase chain reaction was subsequently performed using quantitative PCR (qPCR) was performed on a CFX96 Touch Real‐Time PCR System (Bio‐Rad) with SsoAdvanced Universal SYBR Green Supermix (Bio‐Rad, Cat. #1725271) or Quantitec SYBR Green PCR (Quiagen Cat. #204343). Commercially available validated primers for interleukin (IL)‐33 (QuantiTect, Cat. #QT00897092), LCN2 (Bio‐Rad, Cat. #qCfaCIP0002507), IL‐4 (Bio‐Rad, Cat. #qCfaCIP0015120), IL‐6 (Bio‐Rad, Cat. #10042976), COX1 (Bio‐Rad, Cat. #qCfaCEP0005352), COX2 (Bio‐Rad, Cat. #qCfaCEP0005353), tumour necrosis factor‐alpha (TNF‐α) (Bio‐Rad, Cat. #qCfaCEP0011393), PPAR‐α (Bio‐Rad, Cat. #qCfaCED0030703), TRPV1 (Bio‐Rad, Cat. #qCfaCEP0019089), GPR55 (Bio‐Rad, Cat. #qCfaCED0037760), glyceraldehyde‐3‐phosphate dehydrogenase (GAPDH) (Bio‐Rad, Cat. #qCfaCED0037760) and ACTG1 (Bio‐Rad, Cat. #qCfaCEP0016209) were used. The mRNA level fold change was determined using the 2‒∆∆Ct data analysis method.^30^ Normalisation was performed using GAPDH as an internal control. The stability of this reference gene in canine mammary gland specimens has been previously validated by Etschmann et al.,^31^ and its reliability was confirmed in our samples by the consistency of cycle threshold (Ct) values across experimental groups (mean Ct variation < 0.5).

### Cell cycle analysis

After 24 h exposure to 33 µM CBD (IC_50_) or vehicle control, 1 × 10^6^ cells were harvested and stained according to manufacturer's protocols ^32^ with FxCycle PI/RNase Staining Solution (Invitrogen Thermo Fisher, Cat. #F10797). Samples were acquired on an Attune NxT flow cytometer (Thermo Fisher Scientific).

### Enzyme‐linked immunosorbent assay

DuoSet enzyme‐linked immunosorbent assay (ELISA) kits R&D System were used to evaluate cytokines concentration for TNF‐ɑ (Cat. #DY1507), IL‐8 (Cat. #DY1608), IL‐6 (Cat. #DY1609) and IL‐17A (Cat. #DY5848) following the manufacturer's instructions.^28^

### Statistics

Data represent the mean ± standard error of the mean from three independent biological replicates (*n* = 3 dogs), each assayed in technical triplicate to ensure experimental consistency. Statistical tests were performed in GraphPad Prism v10.0 (GraphPad Software). Normality of distribution was verified with the Shapiro–Wilk test. Student's *t*‐test was employed for comparisons between two groups, while one‐way ANOVA analysis was used for comparisons involving more than two groups with a single variable, followed by Tukey's test. Statistical significance was determined as a *p*‐value of less than 0.05.

## RESULTS

### Histological analysis

**FIGURE 1:**
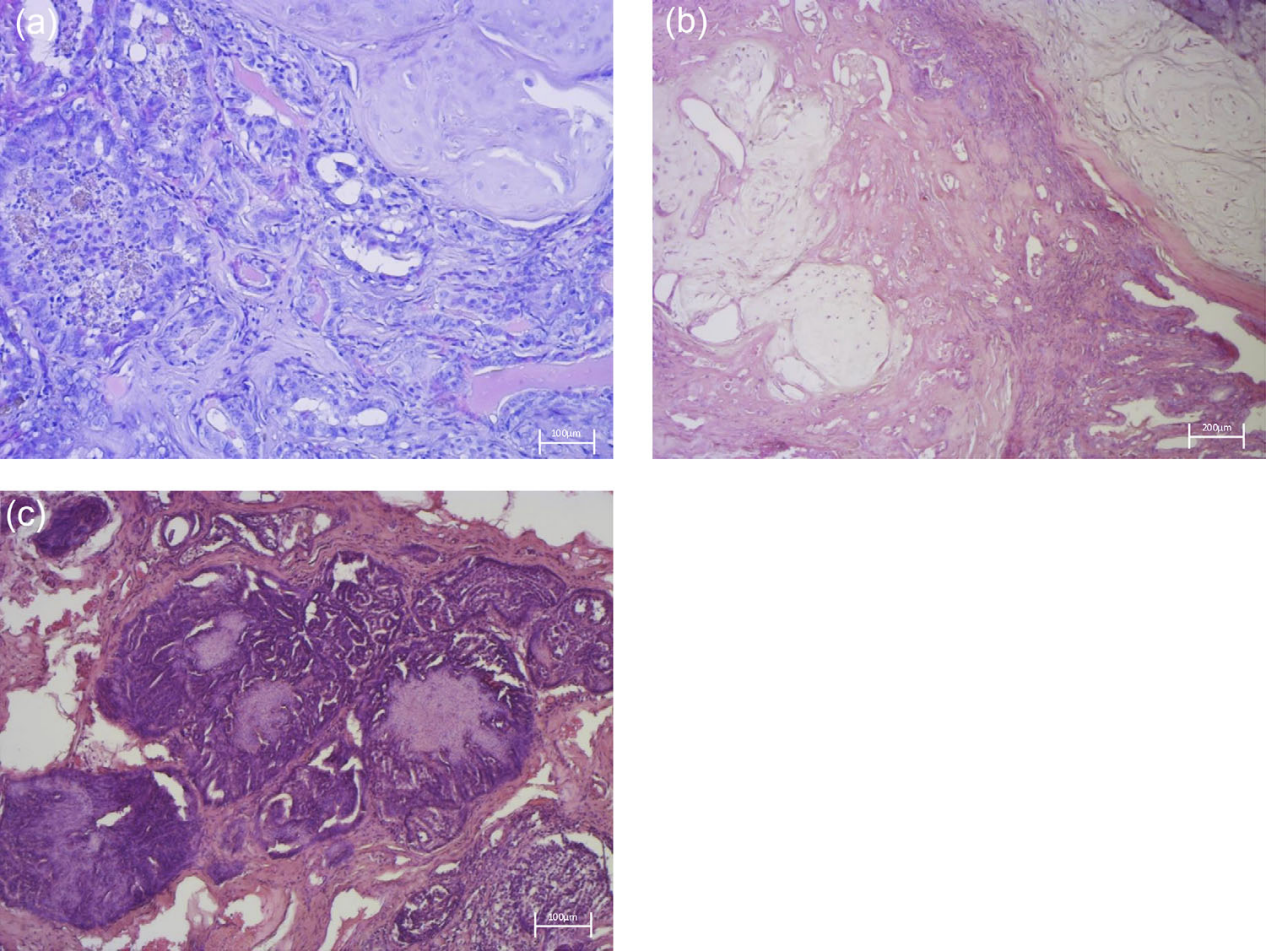
Histopathological characterisation in tissues of canine mammary carcinoma (CMC). (a) Malignant epithelial cells showing solid or tubulopapillary growth patterns, hypochromic nuclei and prominent nucleoli (haematoxylin and eosin [H&E], 20×). (b) Biphasic tumour structure showing neoplastic epithelial cells associated with benign myoepithelial components and metaplastic cartilage (H&E, 10×). (c) Area of comedocarcinoma characterised by neoplastic cells surrounding central necrotic and apoptotic zones (H&E, 10×).

Histological examination shown in Figure 1 revealed pleomorphic, malignant epithelial cells associated with a benign myoepithelial component and metaplastic cartilage (which exhibit no atypia) supported by a fibrovascular stroma. Malignant epithelial cells were arranged in tubules; they showed hypochromic nuclei, large single nucleoli and eosinophilic cytoplasm with distinct margins. In some areas, neoplastic cells surrounded necrotic zones containing cellular debris and apoptotic bodies (comedonecrosis). The malignant epithelial cells at the periphery showed a pattern of growth solid or tubulopapillary, moderate anisokaryosis and mitotic activity than 8 per 2.37 mm. Based on the histological features highlighted, it was possible to make a diagnosis of carcinoma mixed type with areas of comedocarcinoma.^33^

### Microscopic morphology of canine mammary primary cell carcinoma

**FIGURE 2:**
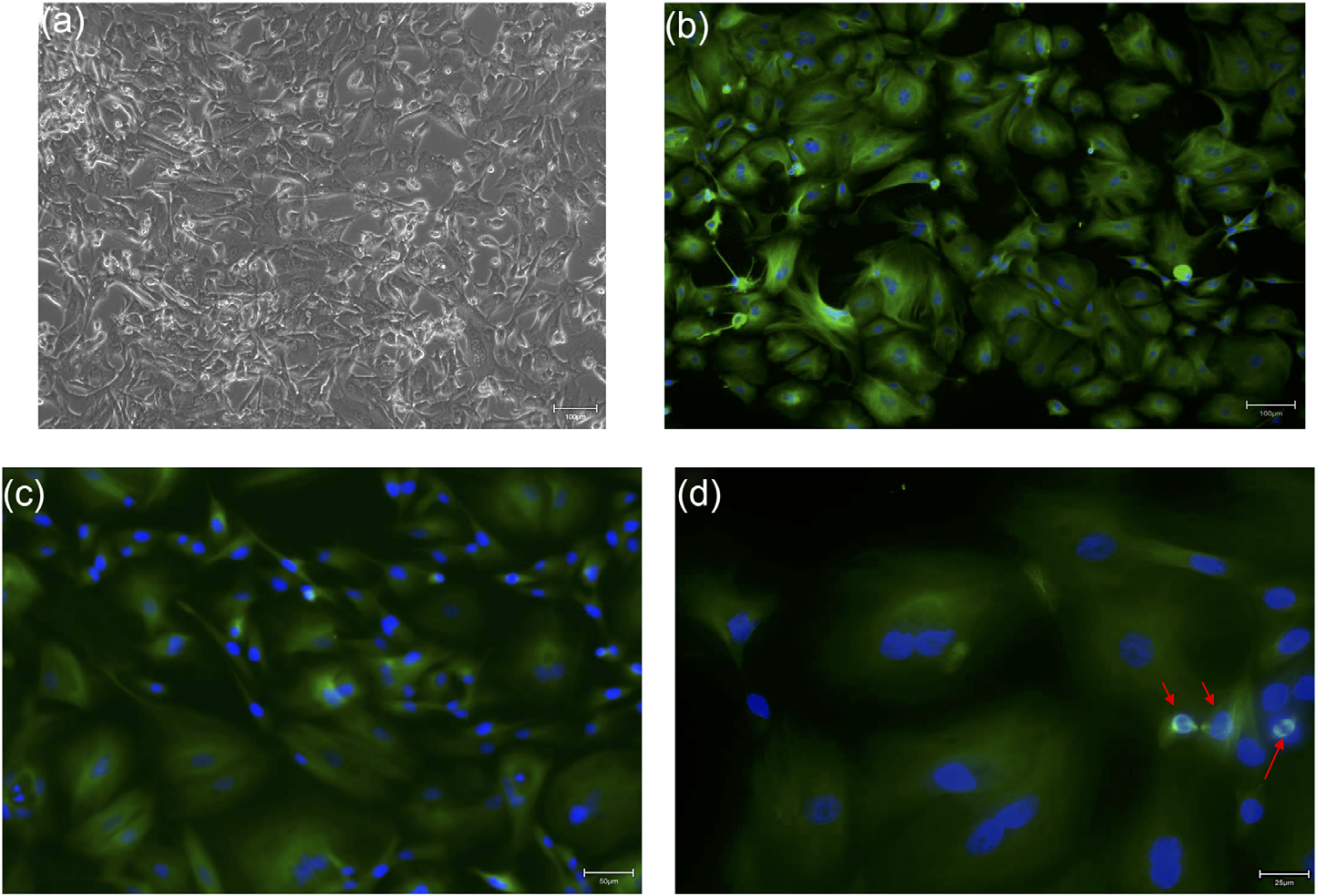
Morphology and β‐tubulin immunofluorescence of canine mammary carcinoma (CMC) cells. (a‒c) Phase‐contrast image (P1) showing mixed spindle and polygonal cell shapes. Immunofluorescence: β‐tubulin (green) decorates the cytoskeleton of proliferating cells; nuclei counterstained with 4′,6‐diamidino‐2‐phenylindole (DAPI) (blue). (d) High‐magnification view highlighting β‐tubulin‐positive mitotic spindles (arrowheads).

As shown in Figure 2, phase‐contrast microscopy revealed a heterogeneous population comprising spindle‐shaped and polygonal epithelial‐like cells (Figure 2a), consistent with the biphasic nature of the original tumour. This morphological heterogeneity persisted from the first sub‐culture (P1) through late passages (≥P6), while cells maintained stable, rapid proliferation. Immunofluorescence for β‐tubulin—a cytoskeletal marker linked to high‐grade mammary malignancies and showed intense cytoplasmic staining and highlighted mitotic spindles, confirming the high mitotic activity of the culture (Figure 2b‒d).^25^, ^34^

### Basal inflammatory profile of primary CMC cell

**FIGURE 3:**
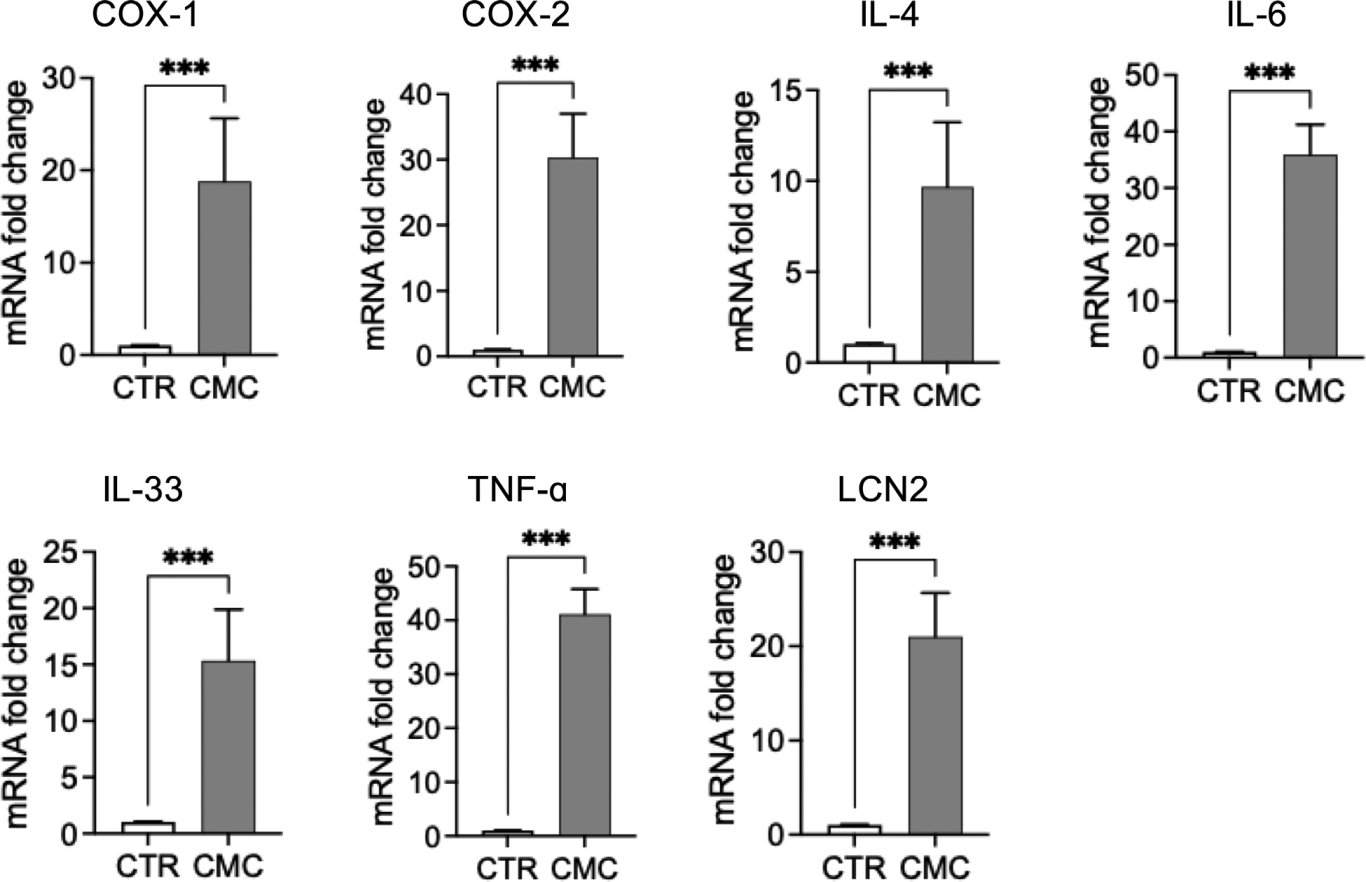
Inflammatory and regulatory gene expression. mRNA fold change of COX1, COX2, interleukin (IL)‐4, IL‐6, IL‐33, tumour necrosis factor‐alpha (TNF‐α) and LCN2 in the canine mammary carcinoma (CMC) group relative to the healthy control (CTR) group by reverse transcription quantitative polymerase chain reaction (RT‐qPCR). The data represent mean ± standard error of the mean (SEM) of independent biological replicates. Statistical significance: ^***^ *p* < 0.001 versus CTR.

**FIGURE 4:**
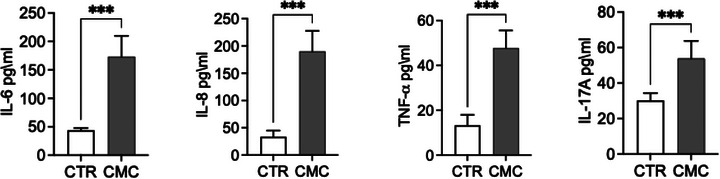
Evaluation of the pro‐inflammatory cytokines interleukin (IL)‐6, IL‐8, tumour necrosis factor‐alpha (TNF‐α) and IL‐17A in canine mammary carcinoma (CMC) cells expressed in pg/mL by enzyme‐linked immunosorbent assay (ELISA) test. The data are presented as mean ± standard error of the mean (SEM). Statistical significance versus control: ^**^ *p* < 0.01; ^***^ *p* < 0.001; ^****^ *p* < 0.0001; ns = not significant.

We evaluated the expression of genes involved in inflammatory and immune response pathways, including COX‐1, COX‐2, IL‐4, IL‐6, IL‐33, TNF‐α and LCN2 (Figure 3). Both the constitutive enzyme COX‐1 and its inducible isoform COX‐2 were significantly upregulated in CMC tissues, where they are associated with disease progression and the maintenance of a pro‐inflammatory state. Notably, while COX‐1 expression was significantly increased, COX‐2 exhibited an even greater upregulation, supporting the presence of an active inflammatory phenotype in this tumour type. To further characterise the inflammatory and immune landscape of CMC cells, we analysed the expression of key cytokines, including IL‐4, IL‐6, IL‐33, and TNF‐α, known to orchestrate tumour‐associated inflammation. Interleukin‐6 and TNF‐α, two major mediators of inflammatory signalling and immune modulation, were significantly elevated at the transcriptional level. In addition to these cytokines, IL‐4 recognised for its role in shaping the tumour microenvironment and influencing immune polarisation—was also significantly upregulated in tumour tissues. Furthermore, we observed markedly increased mRNA levels of IL‐33 and LCN2, both implicated in tumour progression through their involvement in immune cell recruitment as well as in the regulation of proliferation, migration and invasion of tumour and mesenchymal cells. Consistent with the transcriptional findings, analysis of culture supernatants revealed significantly higher concentrations of IL‐6, IL‐8, TNF‐α and IL‐17A in primary CMC cultures compared with control mammary epithelial cells (Figure 7a). These data confirm that CMC cells exhibit not only an activated inflammatory gene expression profile but also a corresponding pro‐inflammatory secretory phenotype (Figure 4).

### Expression of endocannabinoid‐related receptors

**FIGURE 5:**
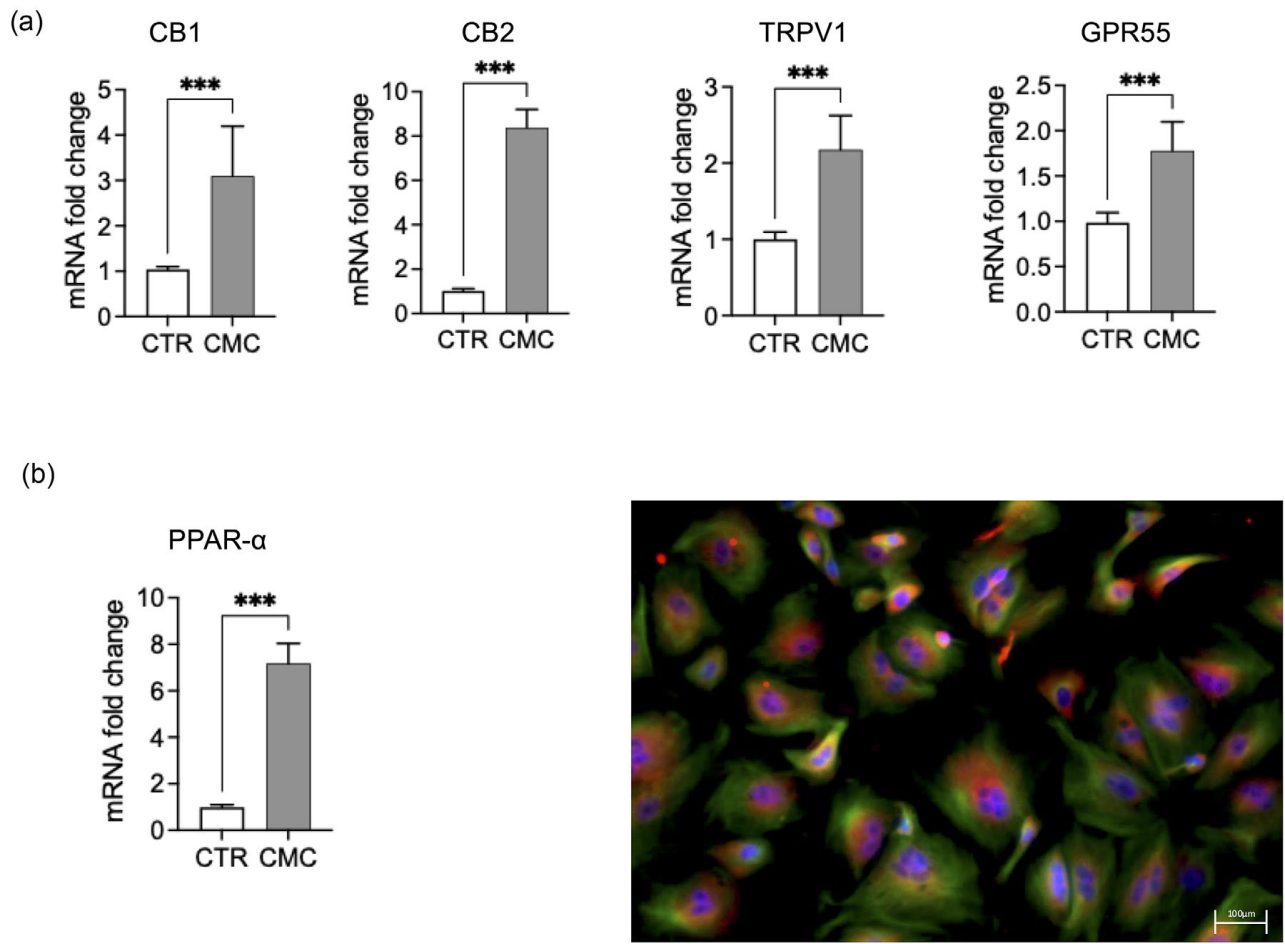
Expression of cannabinoid receptors and peroxisome proliferator‐activated receptor alpha (PPAR‐α) in canine mammary carcinoma (CMC) group. (a) Reverse transcription quantitative polymerase chain reaction (RT‐qPCR) analysis showing mRNA fold change of CB1 and CB2 receptors compared to control (CTR), measured by RT‐qPCR. (b) Representative immunofluorescence image of cells stained for PPAR‐α and 4′,6‐diamidino‐2‐phenylindole (DAPI) (blue staining) to identify cells nuclei. Quantification of PPAR‐α nuclear localisation expressed as percentage of positive nuclei. The data are presented as mean ± standard error of the mean (SEM). Statistical significance versus control: ^***^ *p* < 0.001.

Gene expression analysis revealed a significant upregulation of multiple endocannabinoid‐related receptors in CMC cells compared with matched control mammary epithelium (Figure 5). mRNA levels of CNR1 (CB1) and CNR2 (CB2) were markedly increased in CMC samples. Similarly, the non‐classical cannabinoid‐related receptors TRPV1 and GPR55 showed significant overexpression relative to controls (Figure 5a). In addition, PPAR‐α mRNA expression was significantly elevated in CMC cells (Figure 5b). Immunofluorescence analysis confirmed PPAR‐α protein expression and demonstrated increased nuclear localisation in tumour cells compared to controls, supporting transcriptional activation of this pathway.

### Anti‐proliferative effects of CBD: viability and cell cycle analysis

**FIGURE 6:**
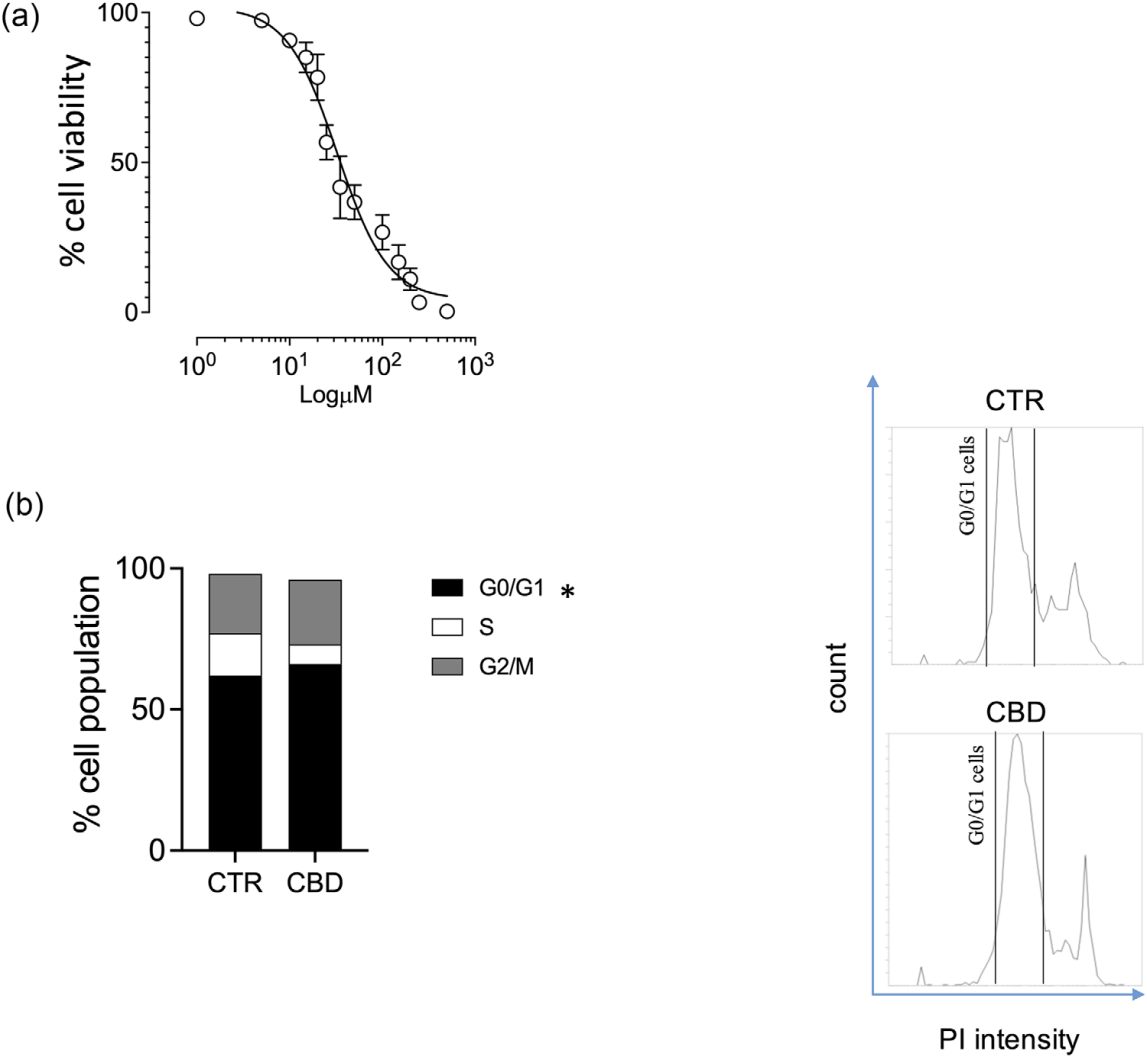
Effects of cannabidiol (CBD) on cell viability and cell cycle progression. (a) Dose‒response curve showing the effect of CBD treatment on cell viability. The curve was fitted using a four‐parameter logistic model to determine the half‐maximal effective concentration (EC_50_). (b) Flow cytometry histograms showing cell cycle analysis after propidium iodide (PI) staining. The top panel represents the untreated control (CTR), while the bottom panel shows the cell cycle profile after CBD treatment, highlighting an increased cell population in the G0/G1 phase. The G0/G1 in the flow cytometry histograms is indicated by the gate in each representative plot to show the percentage of cells in the quiescent/early interphase. Bar graph quantifying the distribution of cells in different cell cycle phases (G0/G1, S and G2/M) for the control (CTR) and CBD‐treated groups. The data are presented as mean ± standard error of the mean (SEM). Statistical significance versus control: ^*^ *p* < 0.05.

To characterise the antiproliferative activity of CBD, we first generated a 24‐h concentration–response curve, identifying an IC_50_ of 32.63 µM (Figure 6a). We then assessed cell cycle distribution after 24 h exposure to 33 µM CBD. Flow‐cytometric analysis of DNA content revealed a significant increase in the G0/G1 fraction compared with vehicle‐treated cells (Figure 6b), consistent with an antiproliferative effect at this concentration.

### Cannabidiol attenuates inflammatory gene expression and cytokine production in primary CMC cells

**FIGURE 7:**
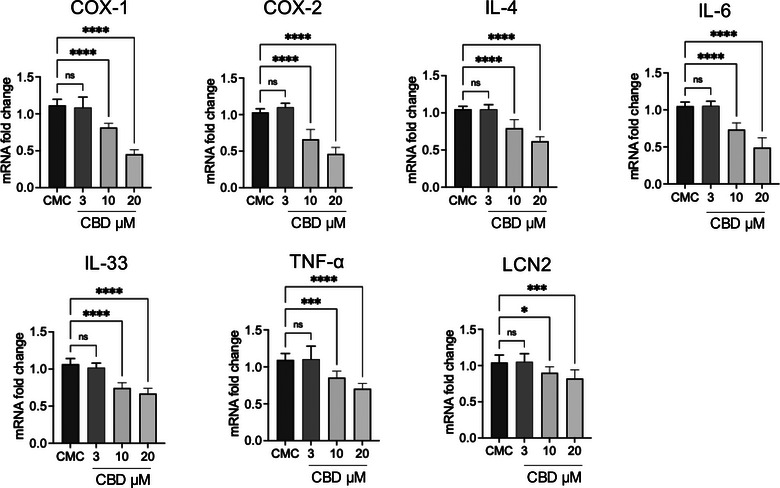
Effect of cannabidiol (CBD) on mRNA expression of inflammatory markers. Reverse transcription quantitative polymerase chain reaction (RT‐qPCR) analysis of COX‐1, COX‐2, interleukin (IL)‐4, IL‐6, IL‐33, tumour necrosis factor‐alpha (TNF‐α) and LCN2 mRNA levels in canine mammary carcinoma (CMC) cells. The data are presented as mean ± standard error of the mean (SEM). Statistical significance versus control: ^*^ *p* < 0.05; ^**^ *p* < 0.01; ^***^ *p* < 0.001; ^****^ *p* < 0.0001; ns = not significant.

**FIGURE 8:**
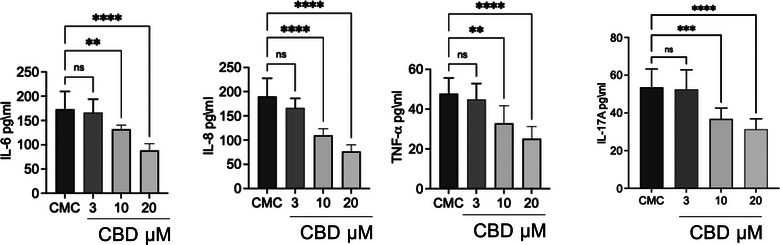
Evaluation of the anti‐inflammatory activity of cannabidiol (CBD) at increasing concentrations (3, 10 and 20 µM) on the secretion of pro‐inflammatory cytokines interleukin (IL)‐6, IL‐8, tumour necrosis factor‐alpha (TNF‐α) and IL‐17A released in the supernatant expressed in pg/mL by enzyme‐linked immunosorbent assay (ELISA) test. The data are presented as mean ± standard error of the mean (SEM). Statistical significance versus control: ^**^ *p* < 0.01; ^***^ *p* < 0.001; ^****^ *p* < 0.0001; ns = not significant.

Treatment of primary CMC cultures with CBD resulted in a consistent, dose‐dependent modulation of inflammatory mediators at both the transcriptional and protein levels (Figures 7 and 8). At the mRNA level in Figure 7, exposure to 3 µM CBD did not produce significant changes compared to untreated CMC cells for most targets, indicating that this concentration is largely sub‐threshold for transcriptional modulation. In contrast, 10 µM CBD significantly reduced the expression of COX‐1, COX‐2, IL‐4, IL‐6, IL‐33, TNF‐α and LCN2. The highest concentration tested (20 µM) induced a further and more pronounced downregulation of all analysed genes, with COX‐2, IL‐6 and IL‐33 showing particularly marked reductions.

Figure 8, on the other hand, highlights the effect of CBD at different concentrations (3, 10 and 20 µM) on cytokine levels in CMC cells supernatant. Cannabidiol dose dependently reduced the levels of IL‐6, IL‐8 and TNF‐α compared to the CMC group. For IL‐17A, the reduction was visible but not statistically significant for concentrations at 3 and 10 µM, becoming significant only at 20 µM.

Consistent with the gene expression data, ELISA analysis of culture supernatants (Figure 8) revealed a dose‐dependent decrease in cytokine release. While 3 µM CBD did not significantly affect IL‐6, IL‐8, TNF‐α or IL‐17A levels, treatment with 10 µM significantly reduced IL‐6, IL‐8 and TNF‐α concentrations. The strongest inhibitory effect was observed at 20 µM, where all measured cytokines, including IL‐17A, were significantly decreased compared to untreated CMC cells. Notably, IL‐17A required the highest concentration to reach statistical significance, suggesting differential sensitivity among cytokine pathways to CBD modulation.

## DISCUSSION

The ECS plays a crucial role in regulating various physiological functions in mammals.^35^ Furthermore, this system is implicated in several pathological states as it serves as an endogenous regulator for restoring both cellular and tissue homeostasis.^36^ In fact, its physiological effects are exploited as therapeutic targets in various diseases including cancer, neurodegenerative diseases such as Alzheimer's disease and Parkinson's disease, chronic pain syndromes and inflammatory disorders.^37^, ^38^, ^39^, ^40^

Recently, the ECS has garnered significant interest in tumourigenesis and cancer therapy.^41^ As is widely recognised, cancer is a complex disease involving various cellular responses and interactions, including dysregulated cell proliferation and apoptosis, angiogenesis, immune evasion and interactions with the tumour microenvironment.^42^ One key feature is increased proliferation and loss of cell cycle control, but inflammatory and immune responses also contribute significantly.^43^

Recent studies have focused on understanding the pathological processes underlying canine mammary tumours, as well as on identifying potential treatments or adjuncts to standard pharmacological approaches.^44^, ^45^ In this context, cannabinoids attract great interest, not only for their well‐known anti‐inflammatory and immunomodulatory properties but also for their potential role in regulating tumour cell proliferation.^46^, ^47^ Moreover, their relatively favourable pharmacological profile allows them to be used in combination with conventional chemotherapy, and in some cases, they exhibit synergistic effects, particularly in glioblastoma, breast cancer and pancreatic cancer models.^48^, ^49^, ^50^

In this study, we examined the role of the ECS in CMC using cells isolated from patients undergoing surgical resection. Histological examination enabled us to identify the specific type of mammary tumour, and the isolated tumour cells stabilised in vitro exhibited phenotypic features consistent with the original histological condition. We then analysed the gene expression of key inflammatory markers, such as COX‐1 and COX‐2, known to be fundamental in shaping the tumour microenvironment through immune cell recruitment and inflammatory responses. Both COX‐1 and COX‐2 were significantly upregulated in tumour tissues. These enzymes are known mediators of inflammation in both human and canine mammary cancers.^51^, ^52^ Notably, in various tumour types, including breast cancer, COX‐2 has been linked to disease progression and often correlates with tumour invasiveness and aggressiveness in both humans and animals.^53^ Accordingly, the use of non‐steroidal anti‐inflammatory drugs, especially selective COX‐2 inhibitors, can have beneficial effects in such cancers.^54^

To study CBD's anti‐inflammatory properties without the interference of significant cell death, dosages were selected based on preliminary cytotoxicity tests. By using concentrations corresponding to a 20% dilution of the CBD stock solution, we ensured that the effects observed on CMC cells were primarily due to biochemical signalling rather than reduced cell viability. Among the most significant pleiotropic pro‐inflammatory cytokines involved in breast cancer progression are IL‐6 and TNF‐α, both overexpressed in this setting.^55^ Interleukin‐4, a pleiotropic cytokine produced by T lymphocytes, acts on various cell types (T and B lymphocytes, monocytes, fibroblasts, endothelial cells and macrophages) and contributes to tumour progression and metastasis by promoting cell survival and proliferation, modulating immune responses towards a tumour‐permissive phenotype and activating nuclear factor‐kappa B (NF‐κB) signalling.^56^, ^57^ Interleukin‐33, a member of the IL‐1 cytokine family, is constitutively expressed in the nuclei of epithelial, endothelial and fibroblast‐like cells.^58^ Under cellular stress, damage or necrosis, IL‐33 is released into the cytoplasm to function as an alarmin, binding its specific receptor ST2.^59^ Interleukin‐33 exhibits pleiotropic roles in inflammatory diseases, particularly in cancer.^60^ Its influence can be either pro‐ or anti‐tumourigenic, depending on tumour type, expression levels, bioactivity and the nature of the inflammatory environment.^61^ Experimental studies have demonstrated that IL‐33 can directly promote tumourigenesis by enhancing proliferation and colony formation in breast cancer cell lines.^32^ LCN2 also emerges as a key player, as it is expressed at high levels in carcinoma tissues.^62^ Over the past two decades, abnormal LCN2 expression has been linked to various pathological conditions, including inflammation and cancer in multiple organs.^63^ Such findings may prove useful in guiding pharmacological interventions in veterinary oncology and identifying new therapeutic targets. Previous studies have demonstrated that components of the ECS are dysregulated in various cancers and are associated with tumour progression, inflammation and patient prognosis.^64^, ^65^, ^66^ In this context, our study expands on these findings by characterising the expression of ECS‐related receptors, together with key inflammatory markers in primary CMC cells, thereby providing further insight into the interplay between ECS signalling and tumour‐associated inflammation.^18^, ^67^

Previous research has indicated that cannabinoid receptors can modulate the growth and development of mammary tumours.^68^, ^69^ Their expression may change within tumours due to both endogenous ECS regulation and interactions with exogenous cannabinoids.^70^ Indeed, in breast tumours, CB1 and CB2 receptor levels often differ from those in healthy tissues, influencing how tumour cells respond to cannabinoids.^71^ Because the ECS has shown anti‐cancer potential,^72^ we assessed CB1 and CB2 receptor expression in CMC cells. Our results revealed elevated CB1 and CB2 expression relative to healthy tissue controls. Although the present study focused on evaluating the global modulation of the ECS, it is important to consider that the ECS contains numerous receptors, including PPAR‐γ, which play a crucial role in mediating anti‐inflammatory effects, such as inhibition of pro‐inflammatory cytokine production, suppression of NF‐κB signalling and reduction of immune cell activation.^73^, ^74^ The effect of CBD on this receptor has been highlighted in several studies.^70^, ^75^ Its modulation by the CBD used in this study was confirmed through its upregulation in these tumour cells.^76^ Both our in vitro assays and molecular analyses confirmed higher levels of PPAR‐α in tumour cells.

The genes for TRPV1 and GPR55 are also increased in dogs with this disease.^77^ The overexpression of TRPV1 and GPR55 receptors reveals the involvement of alternative signalling pathways.^26^, ^70^ Indeed, the TRPV1 receptor, although known for its role in pain perception, is increasingly recognised as a mediator of tumour growth and tissue invasion.^78^ In parallel, the GPR55 receptor has been widely demonstrated to be involved in tumour cell survival and metastasis.^79^

The spontaneous occurrence of CMC also highlights the relevance of this model in a comparative oncology context, as dogs share the same domestic environment and are exposed to similar environmental stressors and pollutants, including heavy metals, which have been associated with cancer development in highly industrialised areas.^80^, ^81^, ^82^, ^83^ In this framework, the modulation of inflammatory and proliferative pathways observed in our study further supports the translational value of targeting the ECS.

Our results demonstrate that CBD exerts a significant antiproliferative effect on CMC cells, characterised by an IC_50_ of approximately 33 µM. This reduction in viability is closely related to a clear modulation of the cell cycle, with a significant accumulation of cells in the G0 phase following exposure to CBD.^84^ Regarding the pharmacological relevance of these concentrations, a frequently debated point in veterinary oncology, it is important to contextualise these findings. Although an IC_50_ of approximately 33 µM exceeds the peak plasma concentrations (Cmax) typically observed in dogs, which range from 0.1 to 2.5 µM according to Di Salvo et al.^85^ Cannabidiol's high lipophilicity and its large apparent volume of distribution should be taken into account when considering the pharmacological relevance of the concentrations tested in vitro.^86^ Although the IC_50_ identified in our model exceeds the peak plasma concentrations reported in dogs, the physicochemical characteristics of CBD suggest that its distribution is not adequately reflected by circulating levels alone. At present, however, direct measurements of CBD concentrations within mammary tumour tissue are unavailable, and dedicated pharmacokinetic studies will be necessary to clarify this aspect.

Importantly, we observed that even at sub‐cytotoxic concentrations (3–10 µM), CBD significantly reduced inflammatory gene expression and cytokine secretion. This indicates that meaningful biological activity occurs at levels below those required to impair cell viability. The overexpression of ECS‐related receptors detected in CMC cells may contribute to an enhanced sensitivity to CBD, potentially creating a signalling context in which inflammatory and proliferative pathways are particularly amenable to modulation.^87^

These findings suggest that ECS upregulation in CMC could represent a therapeutically relevant vulnerability deserving further investigation. In turn, CBD may have therapeutic potential in the treatment of this tumour, acting as multi‐target modulator of the ECS. Although these preliminary results are promising, future studies should clarify the underlying mechanisms of action and evaluate the clinical applicability of these approaches.

## LIMITATIONS

Although these results are encouraging, it is important to acknowledge some limitations. The research used primary cell cultures from a small cohort of three canines. A larger and more diverse sample is needed to accurately reflect the histological and molecular variability of CMC, despite the statistical significance of these findings. Furthermore, although the 2D in vitro model used here is crucial for mechanical characterisation, it cannot accurately mimic the intricate 3D structure of a solid tumour or the systemic immunological interactions found in a living human.^88^

To better understand the molecular interactions between the ECS and inflammatory mediators, future research should focus on more in‐depth mechanistic investigations. To evaluate the safety, pharmacokinetics and therapeutic efficacy of CBD as an adjuvant treatment, it will be crucial to move from in vitro models to controlled clinical trials in canine patients. Furthermore, it should be noted that while gene expression analysis provided robust data on the modulation of the ECS and inflammatory markers, validation at the protein level for receptors and COX enzymes remains a challenge. This is primarily due to the limited availability of high‐affinity antibodies specifically validated or cross‐reactive for canine targets.^89^ Future studies will benefit from the development of more specific proteomic tools for veterinary oncology. Our results demonstrate that CBD modulates inflammation in CMC cells, likely through a mechanism related to the ECS. Although further studies using specific inhibitors are needed to confirm these pathways, this study represents a crucial step in understanding the therapeutic potential of cannabinoids in canine patients.

## CONCLUSIONS

This study provides evidence that CMC exhibits a significant overexpression of CB1, CB2, TRPV1, GPR55 and PPAR‐α, indicating significant upregulation of the ECS. Our data suggest that CMC cells actively modulate these signalling pathways to sustain a robust pro‐inflammatory microenvironment. We have demonstrated that this inflammatory cascade is effectively interrupted by CBD; at non‐cytotoxic levels (10–20 µM), CBD reduces cytokine release and silences key inflammatory genes without compromising overall cell survival. Furthermore, it has been shown that CBD therapy could prevent uncontrolled tumour cell proliferation by inducing cell cycle arrest in the G0/G1 phase.

These findings may also have relevant implications for human health, as CMC shares key molecular and pathological features with human breast cancer. Therefore, the modulation of ECS‐related pathways observed in this study may reflect conserved mechanisms that could be exploited for the development of novel anti‐inflammatory and anti‐tumour strategies in human oncology.

## AUTHOR CONTRIBUTIONS

*Methodology, formal analysis and writing—original draft*: Gianluca Antonio Franco. *Methodology and data acquisition*: Ylenia Marino. *Investigation*: Claudia Rifici. *Formal analysis*: Roberta Fusco. *Supervision and validation*: Rosanna di Paola. *Conceptualisation and supervision*: Salvatore Cuzzocrea. *Data curation and writing—review and editing*: Giuseppe Catone and Cecilia Vullo. *Conceptualisation, supervision and writing—original draft and review and editing*: Enrico Gugliandolo.

## CONFLICTS OF INTEREST

The authors declare they have no conflicts of interest.

## FUNDING INFORMATION

None.

## ETHICS STATEMENT

None.

## Data Availability

There are no additional data available. All data generated or analysed during this study are included in the published article.
